# Speaking the Public’s Love Language: How Engaging with Lay Theories Can Build a Rigorous and Resonant Science of Relationships

**DOI:** 10.1177/10888683261441653

**Published:** 2026-04-25

**Authors:** Emily A. Impett

**Affiliations:** 1 Department of Psychological and Brain Sciences, University of Toronto Mississauga, ON, Canada

**Keywords:** close relationships, lay theories, implicit relationship theories, responsiveness, science

## Abstract

**Academic Abstract:**

Popular lay theories shape how millions of people understand and communicate about romantic relationships, yet they are rarely examined scientifically. Rather than dismissing these frameworks as misconceptions, this review argues that engaging with lay theories can advance a science of relationships that is both empirically rigorous and publicly resonant. Using the love languages as a case study, this review introduces a bidirectional framework in which lay theories inform scientific inquiry and scientific insights are translated back to the public. Engaging with lay theories can refine theory, clarify core relational processes, and reveal contextual and cultural blind spots in lay theories. In turn, examining why lay theories resonate highlights unmet public needs, opportunities for improved scientific communication, and implications for clinical practice, relationship education, and public policy. The result is a science of relationships that is rigorous and resonant with the lived experiences of those it aims to serve.

**Public Abstract:**

Popular ideas about relationships—such as the love languages—shape how millions of people understand love, communicate needs, and decide whether their romantic relationships are working. Yet these ideas are rarely examined by scientists, even though they influence real-life relationship decisions. This article argues that popular relationship theories should not simply be dismissed as wrong, but carefully studied as windows into what people need, value, and struggle with in their relationships. Using the love languages as an example, this review shows how engaging with popular ideas can help scientists improve theories of love, communicate research more clearly, and design guidance that better fits people’s lives and relationships. This review also highlights potential risks of oversimplified frameworks, especially when they ignore cultural context. By building a science of relationships that is both rigorous and relatable, researchers can offer guidance that is more inclusive, useful, and responsive to the public.

## Introduction

Over the past two decades, relationship scientists have built a compelling evidence base showing that romantic relationships are among the most reliable and consequential predictors of psychological well-being, physical health, and longevity (e.g., Proulx et al., 2007; Robles et al., 2014). Yet when people make sense of their own relationships, they rarely turn to scientific models. Instead, they rely on popular theories—such as the “love languages” or “soulmate theories”—that give their experiences meaning and direction. These ideas are not idiosyncratic beliefs but widely circulating cultural scripts, learned through popular books, social media, films, television, relationship advice columns, and online quizzes. For example, more than 30 million people have taken a quiz to discover their love language (Fetters, 2019), and 60% of Americans believe in soulmates (YouGov, 2021). Even constructs long neglected in psychological research, such as interpersonal chemistry (Liepmann et al., 2025; Reis, Regan, & Lyubomirsky, 2022), remain central to how people understand love and attraction.

These ideas are examples of lay relationship theories: informal frameworks that people use to interpret a partner’s feelings, communicate needs, and navigate intimacy. Lay theories provide accessible explanations for why relationships succeed or fail, supply common language for articulating relationship expectations, and shape everyday decisions about how to behave toward romantic partners. Few lay theories have permeated public consciousness to the same degree as the love languages. Chapman’s (2015) book, *The 5 Love Languages*, has sold over 20 million copies, been translated into more than 50 languages, accumulated billions of views on TikTok, and been incorporated into relationship education programs (as cited in Bunt & Hazelwood, 2017). The framework offers a deceptively simple shorthand for how people give and receive affection: five categories of loving behavior—words of affirmation, quality time, acts of service, physical affection, and gifts—a single primary “language” per person, and a clear prescription for relationship success: identify your partner’s language and learn to “speak” it.

The cultural reach of the love languages is striking, but the psychological appeal underlying the framework is not unique. The love languages belong to a wider ecosystem of lay theories that people use to explain relational dynamics, reduce uncertainty, and maintain a sense of coherence and control in their intimate lives. As social and personality psychologists have long observed (e.g., Dweck et al., 1995; Furnham & Henley, 1988; Levy et al., 2006), people routinely rely on intuitive theories to make sense of complex social behavior. In close relationships, these intuitive theories include beliefs about “speaking a partner’s love language,” the idea that men and women “are from different planets” (Gray, 1992), and the belief that relationships succeed or fail based on romantic destiny (Knee, 1998). Although such theories can be reductive—for example, by reinforcing gender essentialism (Carothers & Reis, 2013) or promoting rigid interpretations of compatibility (Knee, 1998)—their cultural persistence reflects a deeper psychological reality: people have an enduring need for intuitive, actionable guidance about love and relationships.

Lay theories matter not only because they are culturally widespread but because they are psychologically consequential and behaviorally impactful. They shape how people navigate relationships—from assessing compatibility in the early stages of a relationship to deciding whether to persist through difficulties or disengage from relationships. For example, research on implicit relationship theories shows that destiny-oriented beliefs—the conviction that relationships are “meant to be”—are associated with lower persistence and greater disengagement in the face of relationship difficulty, in part because challenges are interpreted as evidence of fundamental incompatibility rather than problems to be worked through (Franiuk, Cohen, & Pomerantz, 2002; Franiuk, Pomerantz, & Cohen, 2004; Knee, 1998). Related work in the domain of sexuality shows that whether people believe good sex depends on “finding the right match” or “working at it over time” shapes how they respond to sexual challenges, including whether they persist through difficulties or disengage when desire fluctuates (Maxwell et al., 2017). Extending these patterns beyond ongoing relationships, recent research shows that people who believe in romantic destiny continue to contact and monitor ex-partners online, particularly those who believe their ex-partner is “the one” (Thompson et al., 2025). Together, these findings demonstrate that lay theories do more than organize people’s interpretations of love; they actively guide relationship decisions with lasting consequences.

Despite their widespread influence on how people understand, evaluate, and navigate their relationships, popular lay theories have received relatively little sustained empirical attention in relationship science. This gap reflects a convergence of structural and methodological forces that discourage engagement with popular frameworks. Structurally, theories that originate in self-help or popular discourse often fall outside the incentive structures of academic research, where rewards and recognition tend to prioritize formal theoretical lineages over ideas that circulate widely beyond the academy. Methodologically, lay theories are often metaphorical or narrative in form, making them challenging to operationalize without oversimplification. As a result, psychological science has tended to overlook lay theories or treat them as misconceptions to be corrected, rather than as empirical signals of the psychological concerns and regulatory processes that underlie their widespread appeal.

Yet popular frameworks articulate how people already organize, interpret, and act on relational experience, making them a generative starting point for scientific inquiry. Empirically engaging such frameworks can surface novel questions, constructs, and processes that psychological theories may overlook. Indeed, recent high-quality empirical research testing the assumptions of the love languages (e.g., Chopik et al., 2023; Flicker & Sancier-Barbosa, 2025; Flicker, Sancier-Barbosa, Clemons-Sastanos, et al., 2025; Flicker, Sancier-Barbosa, & Impett, 2025) would not have emerged without taking lay theories seriously as a starting point. Ignoring the frameworks people already use does not diminish their influence; it simply cedes interpretive authority to oversimplified models without the benefit of scientific refinement. Rather than asking whether lay theories like the love languages are “right” or “wrong,” I ask what can be learned by engaging them empirically and theoretically. When a lay theory achieves widespread cultural uptake, it provides a window into how people make sense of intimacy in everyday life and an opportunity to align relationship science with lived relational experiences.

Although this review offers an approach for engaging with lay theories writ large, I focus on relationship-relevant lay theories and use the love languages as a sustained case study. The love languages are uniquely suited for this purpose: they are among the most widely adopted relationship frameworks in contemporary culture, they make clear, testable claims, and they exemplify the broader strengths and weaknesses of many lay theories, including simplicity, categorical structure, and the use of vivid, intuitive metaphors. The five love-language categories map onto well-established constructs in relationship science: quality time (e.g., shared novel experiences; Aron et al., 2022), words of affirmation (e.g., gratitude; Algoe, 2012), physical touch (e.g., affectionate touch; Jakubiak & Feeney, 2019), acts of service (e.g., instrumental support; Feeney & Collins, 2014), and gift-giving (e.g., gifts; Komiya et al., 2019). Crucially, this correspondence allows the love languages to function as a structured, ecologically valid entry point for examining how lay concepts align with—and diverge from—established models in relationship science.

At their core, the love languages formalize a set of intuitive assumptions—that affection can be sorted into a small number of discrete categories, that individuals have relatively stable preferences for how they want their partner to express love, and that relationship success depends on aligning behavior with those preferences (Chapman, 2015). Paradoxically, these assumptions are precisely what make the framework both culturally compelling and scientifically generative. A growing body of empirical work demonstrates that expressions of love extend well beyond five categories, that most people do not possess a single, stable “primary” way of giving or receiving love, and that partners are no more satisfied when love is expressed in a preferred “language” than when it is expressed in other ways (see Impett et al., 2024, for a review; Flicker, Sancier-Barbosa, Clemons-Sastanos, et al., 2025; Flicker, Sancier-Barbosa, & Impett, 2025; Flicker & Sancier-Barbosa, 2025). Rather than undermining the value of the love languages as a case study, these findings illustrate why they are so useful for theory building: they reveal a systematic gap between how people intuitively conceptualize love and the dynamic, context-sensitive processes—such as responsiveness (e.g., Reis, Clark, & Holmes, 2004; Reis, Itzchakov, Lee, & Ruan, 2022; Reis & Shaver, 1988) and need fulfillment (LaGuardia et al., 2000; see Knee et al., 2013, for a review)—that relationship science identifies as central to relationship quality.

The goal of this review is therefore not to provide an exhaustive review of empirical research on the love languages, nor to endorse or dismiss them, but to use them as a case study for showing how sustained engagement with a single, influential lay theory can reveal general principles for how relationship science might productively engage with lay theories more broadly. I propose that engaging lay theories can advance relationship science through two complementary pathways: (1) a lay theories → science pathway, in which widely adopted lay theories are empirically tested to refine constructs, expose conceptual blind spots, and identify neglected relational processes; and (2) a science → lay understanding pathway, in which theoretical and empirical advances are translated back into accessible, culturally resonant models that shape how psychological insights circulate beyond academia. If relationship scientists want to help people build stronger and more satisfying relationships, it is essential to engage with the frameworks people already use to interpret and manage their relational lives. Doing so allows the field not only to test and refine these theories, but also to design public-facing models that preserve intuitive appeal while increasing conceptual and empirical precision. The result is a science of relationships that is empirically rigorous, publicly meaningful, and resonant with the lived experiences of those it aims to serve.

## Why Lay Theories Endure: Psychological Features That Make Them Sticky

To understand why lay theories like the love languages gain traction, it is necessary to examine the psychological features that make them compelling in the first place. Some lay theories endure not because they describe relationships with precision but because they offer vivid metaphors, clear and actionable guidance, and a sense of hope that relationships can be understood and improved. These features satisfy core motivational and cognitive needs by reducing uncertainty, supporting agency, and offering orienting frameworks that help people make sense of and navigate their relationships.

### Vivid, Intuitive Metaphors

Popular theories often provide vivid, intuitive metaphors that aim to simplify complex dynamics. Metaphors shape how people conceptualize abstract experiences, structure their understanding of the world, and guide how they diagnose problems and identify possible solutions across a range of life domains (Landau et al., 2010). Metaphoric framings of love can powerfully shape how people experience and make sense of relational challenges. For instance, framing love as a union between two halves—a metaphor echoed in “soulmate” narratives (Franiuk, Cohen, & Pomerantz, 2002; Franiuk, Pomerantz, & Cohen, 2004)—leads people to perceive conflict as more threatening to relationship quality than framing love as a journey that two partners navigate together (Lee & Schwarz, 2014). Such findings illustrate that the metaphors embedded in lay theories are not merely linguistic devices but function as intuitive frameworks of love that shape meaning-making and emotional responses in relationships. By compressing diffuse relational information into concrete schemas, metaphors reduce uncertainty and organize how people understand relational experiences, making complex dynamics feel legible.

The language metaphor resonates because it frames relational misfires as translation problems rather than relational failures. Instead of asking whether a partner is understanding, responsive, or meeting one’s needs—constructs that require holistic judgments (e.g., perceived partner responsiveness, Reis, Clark, & Holmes, 2004; need fulfillment, LaGuardia et al., 2000), the metaphor invites a simpler diagnosis: partners may simply be expressing care in different ways. This framing can validate people’s experiences (“We care, but we are just missing each other”) and suggests a clear course of action (“I can learn to speak your language”), transforming relational difficulties into solvable coordination problems rather than indicators of intractable incompatibility. These insights highlight why metaphors are so effective for translating complex ideas into intuitive and emotionally resonant insights. Rather than abandoning metaphors, scientists can develop metaphoric frameworks that preserve intuitive appeal while aligning more closely with empirical evidence on the role of responsiveness and need fulfillment in relationships.

### Simplicity and Practical Appeal

Beyond shaping interpretation, popular theories gain traction because they offer clear, usable guidance for navigating relationships. The love languages provide a straightforward prescription: identify your partner’s preferred love language and try to “speak” it. Although this advice oversimplifies the complexities of relational dynamics, its core message—pay attention to what makes your partner feel the most loved—can increase awareness of a partner’s needs and motivate effortful care. The framework’s appeal is further enhanced by its intuitive logic. Most people can readily recall moments when they felt especially “known” or “seen” by a partner (e.g., Reis, Lee, et al., 2018), and the five categories provide a ready-made vocabulary for articulating those experiences. For example, a person might notice that they feel most supported when their partner offers to help with household tasks, or most connected during uninterrupted conversation. This kind of labeling can feel validating because it gives people a name for how they want to be cared for, even when the categories compress a range of emotional needs—such as validation, security, and feeling valued—into simplified labels. When couples use the love languages as a starting point for discussing what makes each partner feel cared for—or as a prompt to try new ways of expressing affection—the resulting intentionality may strengthen their relationship, even without strict adherence to a partner’s “preferred” language (Pagan et al., 2025).

### Motivational Promise

Beyond their practical appeal, lay theories exert enduring influence because of their motivational promise. These frameworks do more than label relationship experiences; they reassure partners of their capacity to understand one another and improve their relationships. The metaphor of a “language,” in particular, frames love not as an ineffable mystery but as a learnable skill. In moments of relational uncertainty, the love languages can foster feelings of competence (“If I learn to speak your language, I can fix this”), predictability (“My partner will feel loved if I follow this formula”), and optimism (“Love can be decoded and repaired”). Psychologically, these experiences help restore a sense of efficacy and connection while providing a structured form of agency that supports core motivational needs emphasized by self-determination theory (Deci & Ryan, 2000). Conceptually, they operate through relational hope by fostering both agency and perceived pathways toward improved future outcomes (Shimshock & Le, 2022). This motivational scaffolding helps explain why such theories are not only cognitively sticky but also emotionally resonant.

## A Bidirectional Framework for Engaging Lay Theories

The psychological stickiness of lay theories poses a central challenge for relationship science. When lay theories shape how people think, feel, and act in their relationships—even when they are empirically imprecise or incomplete—the question is not whether scientists should engage with them, but how. To address this challenge, I propose a bidirectional framework that treats lay theories as both a source of empirical questions and a vehicle for translation. As depicted in Figure 1, the framework distinguishes two complementary and mutually reinforcing pathways—a lay theories → science pathway, through which culturally meaningful, lay frameworks sharpen scientific inquiry, and a science → lay understanding pathway, through which scientific insights are translated back into more accurate, flexible, and actionable insights into relationships. This approach aligns with a broader dual imperative increasingly recognized across the behavioral sciences: generating knowledge that is both scientifically rigorous and publicly resonant (e.g., Contera, 2021; Kazdin, 2008).

**Figure 1.**
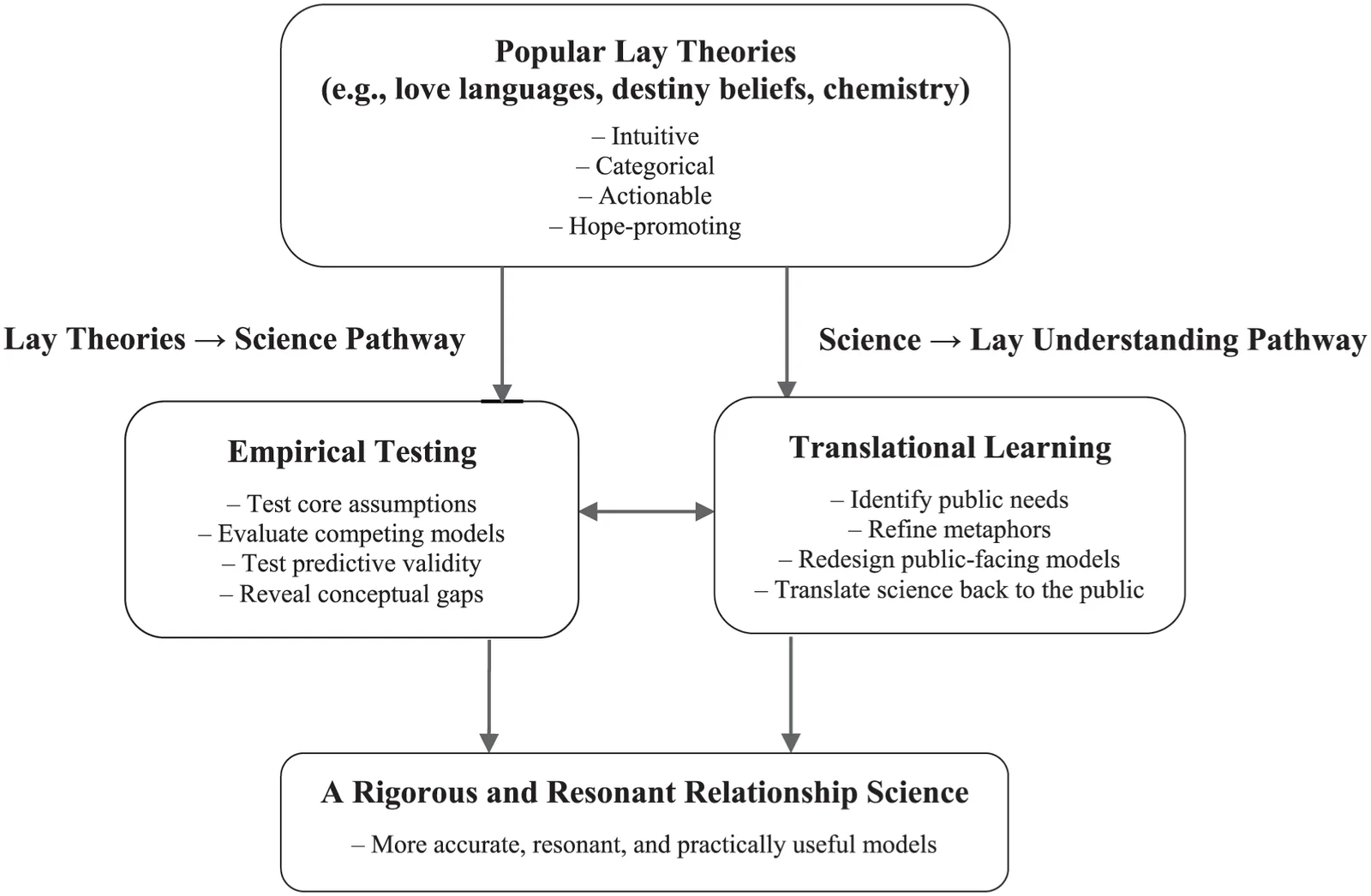
Bidirectional framework of empirical testing and translational learning in relationship science.

In this bidirectional framework, the lay theories → science pathway treats popular frameworks as subjects of serious empirical inquiry rather than as misconceptions to be debunked. When a lay theory achieves widespread cultural uptake, it signals which relational experiences, concerns, and explanations feel most salient in people’s everyday lives. Through this pathway, researchers subject popular models to systematic investigation—testing their core assumptions, evaluating their predictive claims, comparing them with established theories, and identifying where intuitive categories diverge from relational processes documented in the literature. Research examining Chapman’s claims about behavioral matching and relationship satisfaction (e.g., Chopik et al., 2023; Flicker & Sancier-Barbosa, 2025; Flicker, Sancier-Barbosa, Clemons-Sastanos, et al., 2025; Flicker, Sancier-Barbosa, & Impett, 2025) exemplifies this approach, showing how empirical engagement with a lay theory can refine constructs, challenge widely held assumptions, and reveal conceptual blind spots that might otherwise remain obscured.

The science → lay understanding pathway, by contrast, treats translation itself as a scientific task. In this pathway, advances in relationship science are rendered into accessible, culturally resonant forms that shape how people understand and navigate their relationships. Rather than merely correcting inaccuracies, this approach preserves what makes popular frameworks usable—clear metaphors, intuitive structure, and motivational force—while embedding more process-oriented, evidence-based principles. In doing so, scientific insights can refine public metaphors, shift attention from static categories to dynamic responsiveness, and generate public-facing models that more accurately reflect how intimacy is enacted, interpreted, and sustained in everyday life. Critically, these two pathways are not sequential but reciprocal: empirical engagement sharpens and constrains popular theories, while the cultural prominence of lay frameworks draws scientific attention to the questions, meanings, and relational challenges that matter deeply to people. As illustrated in Figure 1, lay theories inform scientific inquiry, and scientific advances, in turn, shape more rigorous and resonant public models of love.

To illustrate how this bidirectional framework operates beyond the love languages, consider lay theories of romantic destiny—the belief that relationships are “meant to be” (e.g., Knee, 1998; Knee & Petty, 2013). In the lay theories → science pathway, destiny beliefs or “soulmate theories” function as empirical signals rather than as a theory to be evaluated. Their widespread cultural presence directs scientific attention to core relational concerns—uncertainty, commitment, and the interpretation of difficulty—and motivates research on how beliefs about fate versus effort organize partners’ responses to challenge. Empirical work shows that stronger destiny beliefs are associated with more rigid interpretations of conflict and a greater likelihood of disengagement when relationships feel difficult, whereas growth-oriented beliefs predict persistence and more adaptive problem solving (Knee, 1998; Knee & Petty, 2013). Here, the scientific contribution is not to replace the lay theory but to identify the psychological processes through which it shapes behavior and outcomes. In the science → lay understanding pathway, these findings can then be translated back into public-facing frameworks that preserve the intuitive appeal of destiny narratives while revising their implications. Rather than framing ease or difficulty as evidence that a relationship is (or is not) “meant to be,” translational models can make visible how beliefs about compatibility and effort shape emotional reactions, attributions, and relationship decisions—highlighting when reliance on destiny beliefs may undermine repair and when reframing difficulty as part of relational growth may support persistence. At the same time, the continued cultural appeal of destiny beliefs feeds back into scientific inquiry, signaling that questions of fit, effort, and commitment remain psychologically central to how people think about love.

### Lay Theories → Science Pathway: What Relationship Scientists Gain by Testing Lay Theories

The lay theories → science pathway in Figure 1 illustrates the role of empirically testing popular relationship frameworks in advancing scientific theory. Lay theories function as a generative lens for identifying where people’s intuitive models of relationships diverge from empirically supported accounts of relational processes. By systematically examining these gaps, empirical engagement with lay theories can advance relationship science in several ways.

First, examining popular frameworks clarifies how people mentally represent love and intimacy, revealing gaps between categorical, behavior-based folk models and the multidimensional, context-sensitive processes emphasized in relationship science. Second, lay theories illuminate how people intuitively explain relationship success and failure, highlighting where relational outcomes are attributed to fixed traits or behavioral matching rather than to responsiveness, motivation, and adaptation. Third, systematic engagement with lay theories surfaces contextual variation—cultural, relational, and situational differences in what people experience as meaningful or loving—that scientific models may not fully anticipate. Finally, lay theories expose how simplified public frameworks shape expectations about effort, reciprocity, and obligation, sometimes fostering rigid norms that constrain flexibility and growth. The sections that follow elaborate on each of these contributions, using the love languages as a sustained case study to demonstrate how systematic engagement with a widely adopted lay theory can sharpen constructs, challenge assumptions, and advance relationship science.

#### From Fixed Categories to Flexible Dimensions: What Lay Theories Reveal About How People Conceptualize Love

Empirically engaging lay theories highlights a gap between how lay people mentally represent love—often in terms of fixed categories—and the fluid, multidimensional nature of lived relational experience, revealing where folk models of intimacy diverge from the processes documented in relationship science. Lay theories often rely on fixed categories to make complex relational dynamics intelligible. Although the love languages provide a concrete illustration, the tendency to carve fluid relational experiences into discrete types is a defining feature of lay theories across domains. More broadly, decades of research in social cognition show that people are drawn to dichotomies and typologies because they simplify information processing and support efficient judgment and decision-making (e.g., Fiske, 2010; Fiske & Taylor, 2013). Reliance on categorization helps explain why such frameworks gain and retain popularity. Typologies also provide shared language and identity-relevant labels (“I’m a words-of-affirmation person”) that facilitate coordination and validation—features that help explain their cultural traction even when they misrepresent relational nuance, making them especially consequential targets for empirical evaluation.

Empirical tests of the love languages illustrate both the appeal and the limits of this categorical logic. Although the framework assumes that individuals possess a single, stable preference that defines how they give and receive love, empirical findings consistently show that people resonate with multiple forms of affection simultaneously and that these preferences tend to correlate with one another rather than diverge (e.g., Flicker & Sancier-Barbosa, 2025; Flicker, Sancier-Barbosa, Clemons-Sastanos, et al., 2025). Notably, research has shown that having a clearly defined “primary” love language is associated with *lower* relationship quality and greater uncertainty about a partner’s care (Flicker, Sancier-Barbosa, & Impett, 2025), suggesting that rigid preferences may reflect unmet needs or inflexibility, and highlighting how folk theories can misdiagnose what makes partners feel cared for. This pattern aligns with broader theories of need fulfillment (see Knee et al., 2013, for a review), which emphasize that high-quality relationships satisfy multiple fundamental needs—from security and belonging to growth and autonomy—rather than hinging on one specific type of affection. Similarly, the suffocation model of marriage (Finkel et al., 2014) suggests that modern couples increasingly expect relationships to fulfill higher-order psychological needs like growth and self-actualization, needs that demand ongoing cognitive and emotional investment. A narrow focus on a single preferred mode of affection may therefore fail to address the broader “nutritional” needs of a partnership (Impett et al., 2024).

Where lay theories carve loving behaviors into discrete types, dimensional models treat them as continuous variations in emphasis, reframing love as a shifting pattern rather than a fixed type. Dimensional models retain the usability of lay frameworks while accommodating complexity and change, consistent with contemporary measurement in the study of personality (e.g., John et al., 2008) and attachment (e.g., Fraley et al., 2015). Rather than asking what someone is (e.g., “What is your love language?”), dimensional models invite questions about the relative importance and situational meaning of different forms of affection (“How much does each expression of love matter, and when?”). Because these judgments concern relational contribution rather than frequency alone, this shift redirects attention from categorization to process, allowing researchers and practitioners to examine not only which behaviors are expressed but how those behaviors function within interactions. From this perspective, intimacy depends on how loving behaviors are interpreted and integrated into the ongoing interactional dynamics of a relationship. As a result, the same behavior can have very different meanings across contexts. From a dimensional perspective, what matters is not the category of behavior itself, but how it is timed, interpreted, and integrated into the interaction. For instance, a gift offered without emotional attunement may signal obligation rather than care, whereas verbal affirmation, delivered in response to a vulnerable disclosure and experienced as validating, can complete an intimacy-building exchange. In this way, dimensional models help clarify why some expressions foster emotional connection while others leave relational needs unmet.

Capturing these dynamics requires tools that reflect flexibility rather than enforce rigid typologies. Chapman’s online love languages measure relies on a forced-choice format that requires individuals to identify a single “primary” love language, reinforcing the folk assumption that preferences are discrete, stable, and mutually exclusive. Later adaptations using Likert-type ratings allowed people to endorse multiple forms of affection (e.g., Flicker & Sancier-Barbosa, 2024; Flicker, Sancier-Barbosa, Clemons-Sastanos, et al., 2025; Flicker, Sancier-Barbosa, & Impett, 2025), addressing a key limitation of earlier categorical approaches with greater ecological validity. A more generative approach would move beyond measures that assess a “primary language” altogether toward multidimensional rating systems that capture the relative importance and functional role of different expressions of love across contexts and time. Such tools could reveal the functions different behaviors serve in context—for instance, how physical affection becomes especially meaningful during periods of stress, or how acts of service feel most loving when offered spontaneously by a partner rather than requested. By emphasizing interpretation and situational meaning over categorical classification, this approach preserves the accessibility of lay frameworks while aligning measurement more closely with the fluid, context-dependent nature of relational needs.

#### From Behavioral Matching to Context-Sensitive Responsiveness: What Lay Theories Reveal About How Love Is Interpreted

Empirically engaging lay theories reveals both their limitations and their diagnostic value for understanding how intimacy is interpreted in everyday life. Popular frameworks tend to locate relational success in matching specific behaviors—“speaking” a partner’s love language—rather than in the interactional processes through which those behaviors acquire meaning. Models such as the love languages suggest that people naturally anchor their judgments of love to visible, nameable acts, and that relationship quality hinges on whether partners express affection in the “right” form. This behavioral emphasis is theoretically generative because it foregrounds observable acts of care while also inviting a deeper question: what must these behaviors accomplish to sustain intimacy over time?

Relationship science addresses this question through the concept of responsiveness—the extent to which a partner’s actions convey understanding, validation, and care (Reis, Clark, & Holmes, 2004; Reis, Itzchakov, Lee, & Ruan, 2022). Classic models of intimacy conceptualize responsiveness as unfolding through a sequence of interdependent steps: the expression of a need or desire, a partner’s recognition and interpretation of that signal, the partner’s behavioral response, and the recipient’s evaluation of that response (Reis & Shaver, 1988). Breakdowns can occur at any point in this sequence, meaning that relational difficulties are not reducible to the selection of an incorrect behavior. Instead, they often reflect misalignment in how signals are noticed, interpreted, or responded to in context (e.g., Lemay et al., 2023; see Reis et al., 2017, for a review). Critically, the contrast between lay and scientific models reveals a theoretically important gap: whereas lay accounts tend to locate intimacy in what partners do, scientific models emphasize how relational meaning is constructed through interaction, interpretation, and response.

Responsiveness is inherently dynamic, varying across time, emotional states, and situational contexts. Relationship scientists have shown that these fluctuations matter: day-to-day variation in perceived partner responsiveness predicts corresponding fluctuations in emotional expression, such that people are more willing to share vulnerability on days they feel understood and cared for, and more likely to withhold emotions when responsiveness feels uncertain (Ruan et al., 2020). Over longer time frames, inconsistency in responsiveness—independent of average levels—uniquely predicts increases in attachment anxiety (Günaydın et al., 2021), likely because unpredictable responsiveness undermines people’s abilities to form stable expectations about whether their needs will be met. In this way, fluctuations in responsiveness matter because they shape how partners update expectations about their relationship, transforming momentary changes in responsive care into inferences about reliability, security, and future connection.

Lay theories like the love languages offer a narrative framework for interpreting this variability. Temporary declines in affirmation, time, or touch—the very behaviors treated as diagnostic in the love languages—may be experienced not simply as situational fluctuations, but as evidence that a partner is failing to “speak” one’s language, particularly when responsiveness feels inconsistent. From a scientific perspective, this highlights why people are especially sensitive to instability in connection and why they seek categorical explanations for process-driven change, using simplified labels to manage uncertainty and make sense of variability that unfolds over time. Methodologically, this insight motivates the use of experience sampling and daily diary designs to test whether lay theories amplify the relational significance of short-term fluctuations in responsiveness, converting momentary dips in a partner’s care into broader inferences about their love and commitment. By taking seriously the folk models people use to interpret fluctuations in responsiveness, relationship science can gain a sharper understanding of how intimacy is monitored, interpreted, and sometimes misread in everyday life.

#### From Cultural Scripts to Relational Meaning: How Lay Theories Shape What “Counts” as Love

Lay theories of relationships do not merely describe love; they operate as cultural scripts that encode shared assumptions about what love is, how it should be expressed, and how it is recognized. By circulating widely in everyday life, these frameworks shape the criteria people use to interpret intimacy, specifying which behaviors feel diagnostic of care and which may go unnoticed. Studying lay theories, therefore, reveals not only the behaviors people associate with love, but the culturally embedded meanings and relational ideals that organize how love is perceived and evaluated. When a lay theory gains widespread traction, its popularity signals psychological relevance: it highlights the forms of care people rely on to make sense of their relationships and, in doing so, delineates what “counts” as love within a given cultural context.

Situating lay theories in their cultural origins clarifies which elements reflect potentially generalizable relational processes and which reflect culturally specific constructions of love. Chapman developed the love languages within a relatively homogenous community of married, religious, mixed-gender couples with traditional family values, and this context likely shaped both what he counted as “love” and what went unrecognized. His framework gives little attention to behaviors that support a partner’s autonomy or personal goals outside the relationship—processes linked to greater relationship satisfaction (Knee et al., 2013) and romantic passion (Carswell et al., 2021)—or to the integration of a partner into one’s broader social network, a distinct form of relational investment (Flicker, Sancier-Barbosa, Clemons-Sastanos, et al., 2025) that is especially meaningful for intercultural couples (Naeimi & Impett, 2025).

More broadly, popular frameworks tend to privilege discrete, visible acts of care—such as gifts, verbal affirmation, or practical favors—over diffuse, ongoing forms of relational work that sustain intimacy over time. One important example is emotional labor: the continuous effort involved in anticipating needs, regulating emotions, and maintaining relational harmony (e.g., Erickson, 2005; Hochschild, 1983). This labor includes efforts to shape a partner’s emotional experiences (e.g., soothing distress, amplifying positive affect, and buffering stress, Niven, 2017; Zaki & Williams, 2013). These forms of care are central to how partners feel supported and understood in everyday life, yet they are rarely named or legitimized as expressions of love within simplified lay frameworks. Because such labor disproportionately falls to women in many man-woman relationships (Eagly & Wood, 2012), its relative invisibility within popular models of love reflects not only cultural ideals about intimacy, but also gendered assumptions about which forms of relational effort are expected, noticed, and valued. By privileging countable behaviors over ongoing relational processes, lay theories encode cultural norms about what care should look like and whose work is recognized as loving. Because the love languages emerged within a specific cultural context, they reflect the relational ideals most salient in that setting and offer a map of intimacy that leaves out many forms of care central to other relationships.

Engagement with lay theories also highlights how the meaning of love—and thus which expressions of care are experienced as responsive—shifts across cultural ecologies. This point is especially clear in the case of the love languages, which organize intimacy around discrete, behaviorally explicit expressions of care, implicitly assuming that responsiveness is achieved through visible action and verbal affirmation. Cultural systems, however, shape responsiveness through shared expectations about which behaviors are appropriate, desirable, or even legible as signals of care (Selçuk & Günaydin, 2023). In more independent cultural contexts, self-expression and explicit communication are often treated as the primary routes to responsiveness, whereas in more interdependent contexts, partners may prioritize restraint over expression to preserve harmony and relational balance (Kitayama et al., 2022).

Research on social support illustrates how the same “love language” behaviors can carry divergent meanings across cultural contexts. In more independent settings, practical assistance and unsolicited support—often framed within the love languages as acts of service—can feel intrusive or undermine autonomy (Cutrona & Russell, 2017; see Shaver et al., 2018, for a review). In contrast, in more interdependent contexts, the same behaviors are expected and experienced as signals of care even when needs have not been explicitly voiced (Esiaka et al., 2020; Selçuk & Günaydin, 2023), and experimental evidence shows that such unrequested support predicts well-being as strongly—or more strongly—than emotional support (Wu et al., 2021). Similar reversals emerge in emotional signaling: expressing gratitude—commonly assumed to signal care and responsiveness—increases perceived responsiveness in the United States but decreases responsiveness in Hong Kong (Zhang et al., 2018), where overt praise can violate norms of modesty. Together, these findings underscore a core limitation of behavior-centered frameworks like the love languages: love is not embedded in behaviors themselves, but in the culturally shared meanings through which those behaviors are interpreted.

These cultural dynamics have direct implications for relationship science. A comprehensive science of relationships must account for demographic and cultural diversity (McGorray et al., 2023; Williamson et al., 2021), ensuring that what “counts” as love is not defined by a single cultural script. Models that aim for universality risk flattening the ways intimacy is embedded in local norms, social networks, and interpretative practices. Research using cultural consensus theory (CCT; Romney et al., 1986; Weller, 2007) shows that cultures share broad understanding of what makes people feel loved while also exhibiting meaningful within-culture variability (Heshmati et al., 2017). By tracing where intuitive frameworks align with or diverge from lived relational realities across cultural contexts, researchers can better distinguish core relational processes from culturally specific scripts. In this sense, lay theories are not merely folk beliefs to be tested but empirical tools for expanding the cultural and conceptual reach of relationship science.

#### Revealing Motivational Constraints: How Lay Theories Shape Relational Regulation

Beyond shaping how love is interpreted, lay theories influence how partners regulate effort, obligation, and responsibility within relationships by supplying informal standards for when care “counts.” These simplified models shape everyday judgments about whether sufficient effort has been shown, whether additional investment is warranted, and how unmet needs are understood. Moments in which lay theories constrain relational flexibility are especially informative because they reveal how people decide whether they have done their part—and what follows when they believe they have. For example, someone may believe they have adequately expressed love because they spent “quality time” with their partner or provided an “act of service” by helping their partner with a task, even when these actions do not lead their partner to feel “seen.” Conversely, unmet needs may be taken as evidence that a partner is failing to show love in the “right” way, rather than as a signal that communication or expectations require adjustment. In these moments, rigidity is not merely a limitation of lay theories but a window into the implicit criteria people use to judge when effort is sufficient, which forms of care count, and when responsibility shifts to a partner. By implicitly supplying rules for when love counts and when it should be acknowledged, lay theories shape whether partners persist, withdraw, or assign blame—offering relationship scientists insight into how simplified models interact with core regulatory processes.

The love languages framework offers a clear illustration of how lay theories can shape the ways partners evaluate effort and obligation within relationships. The emphasis on identifying and “speaking” a partner’s preferred form of love aligns with cultural ideals that prize explicit communication, intentional effort, and reciprocity. In many contexts, this logic can foster attentiveness by prompting people to reflect on how their own behaviors affect their partner. However, when adopted rigidly, it could also encourage a transactional mindset (“I expressed love in your language, so you should express love in mine”). Decades of research distinguish communal orientation (Le et al., 2018), characterized by responsiveness to a partner’s needs without tracking inputs and outputs, from exchange orientation, which emphasizes balance, obligation, and scorekeeping (Clark et al., 2010; see Clark & Mills, 2012, for a review). Adopting a transactional, exchange-oriented mindset reliably predicts lower relationship satisfaction over time (Park et al., 2026), suggesting that well-intentioned lay frameworks may sometimes legitimize motivational patterns that undermine intimacy. The diagnostic value of this example lies not in indicting the love languages, but in clarifying what the framework brings into focus about how partners evaluate relational effort. The same acts of care can foster connection or resentment depending on whether they reflect communal or exchange-oriented norms. By examining when and how lay theories promote rigidity, scorekeeping, and giving with “strings attached,” relationship scientists can gain a clearer view of the motivational processes that shape relational trajectories—processes that are difficult to detect when attention is limited to surface behaviors or static preferences.

### Science → Lay Understanding Pathway: What the Public Gains When Relationship Scientists Translate Findings

Relationship science has generated a rich, cumulative understanding of how intimacy, responsiveness, and connection unfold over time. Yet most people do not encounter this knowledge in the forms in which it is generated and evaluated by scientists (e.g., in peer-reviewed journals and textbooks), but through the lay theories, metaphors, and advice that circulate in therapy, relationship education, social media, and everyday conversation. The science → lay understanding pathway therefore centers on a fundamental design challenge: how to translate complex, process-oriented models into frameworks that are psychologically engaging, usable, and meaningful in people’s relationships.

This challenge has implications not only for science communication but also for theory development. Rather than treating translation as downstream dissemination—rendering established findings more accessible after theoretical work is complete—the framework advanced in this review conceptualizes translation as iterative and generative. Engagement with lay theories does not merely extend scientific knowledge outward; instead, it exerts pressure inward, shaping which questions are asked, how constructs are defined, and which models resonate both within and beyond the field. Translation thus functions not as an endpoint of theory, but as a mechanism through which theory can be refined and sharpened. This dynamic is particularly consequential in relationship science, where core constructs—love, commitment, conflict, intimacy—are common in lived experience yet complex in structure. Scientific models emphasize dynamic processes such as responsiveness, mutual influence, and shifting needs over time, whereas public-facing frameworks often condense these dynamics into static types, fixed preferences, or simple rules. The enduring popularity of the love languages makes this tension visible. Despite limited empirical support for its central claims, the framework persists because it offers clear language, vivid metaphors, and a compelling sense that relational difficulties are understandable and actionable.

The lesson is not that scientific rigor should be traded for appeal, but that appeal shapes whether relational insights are noticed, remembered, and put into practice. When translation is poorly designed, central ideas—such as responsiveness as a dynamic, context-sensitive process—remain inaccessible to the very people whose relationships science aims to improve. The work of the science → lay understanding pathway therefore involves designing public-facing frameworks that retain the clarity and motivational promise of lay theories while embedding the flexibility, nuance, and process orientation that characterize relationship science.

In what follows, I outline three translational design strategies for designing public-facing models of love in ways that preserve intuitive appeal while increasing conceptual precision and flexibility. First, public models of love can be expanded to reflect the multidimensional structure of care articulated in relationship science. Second, scientific constructs can be mapped onto everyday language, revealing points of alignment, distortion, and omission between theory and everyday relational discourse. Third, metaphors can be treated as objects of empirical design and testing, given their role in shaping how people allocate effort, interpret change, and regulate intimacy. Together, these strategies can be embedded across clinical practice, relationship education, and public policy.

#### Expanding Public Models of Love to Reflect Multidimensional Care

A first translational strategy involves redesigning public-facing models of love to reflect the multidimensional structure of care emphasized in relationship science. Whereas popular frameworks such as the love languages condense love into a small set of recognizable behaviors, scientific research has converged on a far more differentiated account of how care is enacted and experienced in relationships. Translation therefore, requires, not only correcting inaccuracies in popular models but also expanding what counts as love in public understanding. Recent work illustrates the scope of this gap. Using open-ended reports of what makes people feel loved, Flicker, Sancier-Barbosa, Clemons-Sastanos, et al. (2025) identified a 10-factor structure of loving behaviors that predicts relationship quality more strongly than Chapman’s original typology. Some dimensions overlap with the love languages (e.g., acts of service, gifts, physical intimacy), whereas others capture forms of care that are less frequently foregrounded in popular discourse—such as showing up during adversity, repairing harm, encouraging a partner’s personal growth, and integrating a partner into one’s broader social world. These dimensions align closely with long-standing theories of need fulfillment, communal motivation, and self-expansion, yet remain underrepresented in the simplified frameworks most couples use to understand their relationships.

From a translational perspective, this asymmetry matters because public models do not merely describe love—they organize attention. When frameworks privilege a narrow set of visible behaviors, they shape which forms of care partners notice, value, and negotiate, while leaving other relational processes unnamed and harder to recognize. The central translational challenge is therefore one of recognition: how to render the full range of scientifically established dimensions of care legible within everyday relational understanding. Expanding public models beyond rigid categories toward multidimensional representations of care offers a way to preserve usability while more accurately reflecting how intimacy is built and sustained over time. Translation is not only a matter of expanding scientific models, but of making those dimensions recognizable in everyday relational life. Even when research identifies a rich, multidimensional structure of care, these processes cannot shape how people interpret and navigate relationships unless they are rendered legible through shared language. This creates a second translational challenge: clarifying where scientific concepts map onto everyday relational language—and where gaps remain.

#### Mapping Scientific Constructs and Lay Vocabulary

If love is truly multidimensional, a second translational strategy involves systematically mapping scientific constructs onto the language people already use to make sense of their relationships. The problem is not simply that scientific models are more complex, but that many of their core dimensions lack names. Without such mapping, it remains unclear where scientific models resonate with lived relational discourse and where they fail to register at all. This approach treats vocabulary itself as a site of inquiry, asking which relational concerns are already named in everyday language, which are approximated through metaphor, and which remain largely invisible. One way to do this is by constructing parallel lexicons that track functionally similar concerns across scientific and folk frameworks—linking constructs such as responsiveness, gender role attitudes, and autonomy support to the maxims, metaphors, and shorthand people use to interpret relational experience (e.g., “They were there for me,” “Happy wife, happy life” and “Love shouldn’t hold you back”). Although these vocabularies differ sharply in precision and scope, mapping them at the level of the relational problems they are designed to address makes it possible to identify points of convergence, approximation, and omission between scientific theories and lay understandings of love.

Methodological advances make this kind of mapping increasingly feasible. Large-scale natural language processing of social media discourse (e.g., Seraj et al., 2021), combined with web scraping of advice columns, relationship education materials, and online courses, can be used to quantify which scientific concepts appear in public discourse, which are reframed through lay metaphors, and which are largely absent. Such analyses can reveal not only where relationship science has failed to penetrate public understanding, but also where intuitive language already exists that could serve as an entry point for more nuanced, evidence-based models. Identifying these patterns allows researchers to design tools that preserve the accessibility and motivational pull of folk models while expanding what counts as care. In this way, mapping scientific constructs onto lay vocabulary transforms engagement from a one-way act of correction into a process of conceptual alignment, clarifying not only what people are already trying to understand about their relationships but how scientific insights can meet them where they are.

#### Testing Alternative Metaphors as Translational Tools

Building on efforts to map scientific concepts onto lay vocabulary, a third translational opportunity is to treat metaphor design itself as a subject of empirical inquiry. The central question is not whether relationship science can debunk popular metaphors, but whether it can generate better ones—metaphors that are psychologically engaging while more precisely capturing the dynamic processes that sustain intimacy. Because metaphors structure how people interpret relational experiences, allocate effort, and evaluate success or failure, they function not merely as explanatory devices but as motivational and regulatory frameworks. This makes metaphor design a theoretically consequential site for translation, and one in which relationship scientists are well positioned to contribute.

One particularly generative approach would be to conceptualize love as a menu rather than as a language (Impett et al., 2024). In this framing, expressions of care are understood as options that can be combined in varying proportions across situations, rather than as a single preferred mode to be mastered. The menu metaphor foregrounds balance, choice, and responsiveness: partners must consider which expressions of love to offer, which have been neglected, and what the relationship requires at a given moment. Crucially, menus accommodate shifting needs over time—what nourishes connection during periods of stress may differ from what sustains intimacy during times of growth or stability. A complementary metaphor is to frame love as a dance. Whereas the menu metaphor emphasizes allocation across different forms of care, the dance metaphor foregrounds coordination, timing, and recovery from missteps. From this perspective, relationship quality depends less on selecting the “right” behavior than on partners’ abilities to adjust to each other’s movements, respond to subtle cues, and regain synchronicity when coordination falters. Considered together, the menu and dance metaphors preserve the accessibility of popular metaphors while embedding a more accurate, process-oriented account of how intimacy is regulated in everyday life.

Importantly, these metaphors generate testable hypotheses. Experimental work could examine whether metaphors emphasizing balance versus coordination differentially shape how partners interpret unmet needs, conflict, or asymmetries in effort. For example, does framing love as a menu encourage diversification of care, whereas framing love as a dance promotes flexibility, repair, and tolerance for missteps? Longitudinal research could assess whether adopting process-oriented metaphors predict greater relational stability by shifting focus from rigid matching toward dynamic responsiveness over time. In this way, metaphor testing becomes not merely a dissemination strategy but a generative scientific tool—one that can yield insights into how relational processes are understood, enacted, and sustained. By treating metaphors as subjects of scientific inquiry rather than rhetorical afterthoughts, relationship science can shape the cultural frameworks through which people recognize care and interpret relational challenges.

#### From Translation to Application: Clinical, Educational, and Policy Implications

Translational design becomes most visible—and most consequential—at the point where scientific models are put to work in real-world settings. In this way, applied contexts function not merely as sites of dissemination but as active testing grounds for translation. They reveal whether expanded models of care—rendered in everyday language and supported by resonant metaphors—can meaningfully reshape how people interpret needs, assign responsibility, and regulate intimacy over time. As such, these domains represent the primary pathways through which scientific models of relationships enter everyday relational practice. In what follows, I illustrate how the translational strategies outlined above can be applied across clinical, educational, and policy contexts, highlighting how they loosen rigid categories, bring neglected dimensions of care into view, and promote more flexible, process-oriented understandings of love in the settings where relationships are treated, taught, and governed.

##### Clinical Implications

In clinical settings, clients often frame their needs and frustrations through familiar lay theories such as the love languages (Khaleeli, 2024). Many clinicians already work with these familiar frameworks as accessible entry points for discussing unmet needs, mismatched expectations, and emotional disconnection. Building on this common practice, lay theories can be used more deliberately as translational tools—not as endpoints but as scaffolds for conversations that move beyond fixed categories toward flexible, process-oriented understandings of care. For example, clinicians might invite partners to reflect on which expressions of care feel most meaningful in different contexts, and how the meaning of these behaviors shifts across stressors, life stages, and relational transitions. Therapeutic prompts can also be reframed to emphasize responsiveness and motivation rather than static preferences—for instance, shifting from which behaviors matter (e.g., “Which love language do you prefer?”) to what those behaviors communicate about understanding and care (e.g., “That gesture felt caring because it showed you noticed when I was stressed”).

One way to operationalize this shift is through responsiveness mapping, in which partners identify valued behaviors alongside the intentions, timing, and contextual cues that make those behaviors feel caring. Reviewing recent positive interactions and unpacking whether their impact stemmed from spontaneity, sensitivity to unspoken needs, or moment-to-moment responsiveness can help couples move beyond behavioral “checklists” toward a more flexible understanding of care. Rather than anchoring intimacy to a narrow set of prescribed actions, this process cultivates attunement skills that may be more broadly applicable across situations. Importantly, several established therapeutic approaches already model this kind of translation in practice. Gottman and Silver’s (1999) “love maps” emphasize staying attuned to a partner’s evolving inner world, while Emotionally Focused Therapy fosters emotional safety through structured attachment-focused conversations (Johnson, 2004). Digital interventions such as *OurRelationship* (Doss et al., 2016) and platforms like GottmanCONNECT extend these principles beyond the therapy office, using personalized assessments, guided reflections, and adaptive, evidence-based interventions. Clinicians can work within familiar cultural frameworks while subtly reshaping them, rather than requiring clients to abandon the models they already use. Together, these approaches illustrate how core constructs from relationship science can be translated into emotionally resonant, scalable tools that support real-world relational insight and growth. In this way, translation does not dilute science but becomes a way to make its most powerful insights usable in the settings where relationships are actively negotiated and repaired.

##### Relationship Education

The same translational challenges and opportunities extend to relationship education. In premarital programs, community workshops, and public-facing curricula, educators often confront a gap between what science can offer and what participants experience as accessible, relevant, and actionable. Research underscores this challenge: low-income couples face substantial structural and attitudinal barriers to seeking relationship support, including stigma, skepticism, and limited access (Williamson et al., 2019). Empirically supported interventions fall short when they overlook the structural stressors and contextual barriers couples face (Lavner et al., 2015; see Karney, 2021, for a review). These limitations point to a pragmatic lesson for relationship education: programs are most likely to engage participants when they build on frameworks that feel intuitive, culturally familiar, and aligned with lived experience. The widespread appeal of lay theories illustrates how shared vocabulary and clear narratives can lower barriers to engagement, particularly when formal interventions feel abstract, alienating, or mismatched to context. Yet many relationship education programs remain rooted in heteronormative assumptions, limiting their relevance for couples with LGBTQ+-identifying partners (McGorray et al., 2023). Reflecting this broader disconnect, nearly 90% of studies in a decade-long review of research on romantic relationships and health excluded same-sex couples, even when sexual orientation was not central to the research question (Andersen & Zou, 2015). Taken together, these patterns suggest that the central challenge for relationship education is not a lack of evidence but a failure of translation, particularly in how scientific insights are framed, contextualized, and made meaningful for diverse couples.

One promising way to close this translational gap is to work with the narratives people already use to make sense of their relationships. From this perspective, relationship education can preserve what draws people in—clarity, familiarity, and a sense of hope—while embedding more flexible, process-oriented skills that move beyond fixed preferences or prescriptive ideals. Lay theories resonate strongly in educational settings in part because they are easily woven into personal and shared narratives. Narrative approaches, therefore, offer a particularly promising translational bridge. Research on narrative identity shows that people make meaning through the life stories they tell about their experiences, relationships, and personal growth (McAdams & McLean, 2013). Within romantic relationships, these individual narratives become intertwined as partners co-construct shared stories that organize experience, shape interpretation of events, and foster a sense of “we-ness” (Frost, 2012). Educators can harness these insights by inviting couples to co-construct stories about times they felt cared for in different ways and under different constraints, rather than evaluating relationships against fixed ideals of what love should look like. Reflecting on how expressions of love shift across stressors, identities, and cultural contexts may help couples interpret variability as part of ongoing relational processes rather than as evidence that love is being expressed “incorrectly.” Using popular frameworks as narrative scaffolds—rather than as prescriptive models—allows educators to retain accessibility while introducing nuance, flexibility, and cultural sensitivity. In this way, relationship education moves beyond teaching fixed ideals of love and instead equips couples with tools for interpreting, negotiating, and sustaining connection across changing circumstances.

##### Policy Implications

As social connection is increasingly recognized as a public health priority, translating relationship science has become a matter of policy relevance. Romantic relationships are one of the most consequential and enduring sources of adult social connection (Finkel et al., 2014) and represent a key pathway through which social connection affects health. High-quality romantic relationships promote psychological well-being (e.g., Proulx et al., 2007), regulate health behaviors through mutual influence and social control (e.g., Berzins et al., 2019; Cornelius et al., 2016), and embed individuals within broader networks of social integration and support (Bryant et al., 2001; Sprecher & Felmlee, 2005). When these relational pathways are weakened or disrupted, the consequences extend beyond romantic relationships: chronic loneliness and social disconnection are associated with elevated morbidity and mortality risks comparable to established behavioral risk factors (e.g., Holt-Lunstad et al., 2010; Robles et al., 2014). Together, this evidence suggests that supporting the quality of romantic relationships should be a foundational component of public health policy.

In response, recent public health initiatives have called for coordinated, population-level efforts to strengthen social ties and relational well-being (Public Health Agency of Canada, 2023; World Health Organization, 2025). Holt-Lunstad et al. (2017) have advocated for the development of national guidelines on social connection, modeled after existing guidelines for nutrition and physical activity (Holt-Lunstad, 2023). For romantic relationships in particular, such guidelines could specify behavior-based targets for relational health—regular behaviors that convey responsiveness (e.g., attentive listening and validation; Itzchakov et al., 2022; Reis, Clark, & Holmes, 2004), supportive actions during stress (e.g., emotional reassurance and practical assistance; Feeney & Collins, 2014), and constructive conflict behaviors (e.g., respectful discussion, de-escalation, and repair attempts; Gottman et al., 2015)—and direct funding toward interventions that build these capacities across diverse family forms. As with nutrition or physical activity guidelines, the goal would not be to prescribe a single model of “healthy” relationships, but to provide flexible, evidence-based benchmarks that support sustainable patterns of connection and regulation within intimate partnerships over time.

To be effective at scale, however, policy efforts must rely on forms of translation that resonate with how people already understand romantic relationships. Integrating relationship science into prevention programs, community-based services, and health promotion initiatives will be most successful when scientific principles are embedded within familiar relational frameworks. An underused opportunity is to leverage the cultural reach of widely recognized models—such as the love languages—as entry points for introducing more flexible, evidence-based understandings of care within romantic partnerships. Even when a lay theory’s core claims lack direct empirical support, its public familiarity can function as a powerful vehicle for disseminating scientifically grounded principles, particularly when translation preserves features that drive uptake (clarity, metaphor, and hope) while minimizing the risks of rigid categorization. Viewed through this lens, the science → lay understanding pathway becomes not merely a communication strategy but a policy-relevant design framework for shaping how relational health is defined, measured, and supported at scale.

## Future Directions to Build a Rigorous and Resonant Science of Relationships

This review uses the love languages as a case study to illustrate how popular lay theories organize everyday judgments about love, effort, and care, and how relationship science can engage these frameworks productively. Looking ahead, a central task for the field is to move beyond ad hoc engagement with any single lay theory toward a more principled approach to identifying which lay theories warrant sustained scientific attention—and why. In particular, lay theories are likely to be especially informative because they shape persistence, withdrawal, and repair in close relationships (Franiuk, Cohen, & Pomerantz, 2002; Franiuk, Pomerantz, & Cohen, 2004; Knee, 1998; Maxwell et al., 2017). Lay theories do not all emerge through a single pathway: some originate in clinical practice, others from academic research that later enters popular discourse, and still others from media or social media ecosystems that rapidly amplify intuitively compelling ideas. Survivor bias likely shapes this landscape: many ideas about relationships circulate at any given time, but only those that are especially memorable, emotionally resonant, and easily actionable become culturally dominant. Because these “survivors” are the theories most likely to guide everyday expectations and decisions, they are also the ones most likely to exert downstream effects on how relationships are understood and navigated.

These selection dynamics are further intensified in the contemporary digital landscape, where relationship ideas are generated, circulated, and stabilized at unprecedented speed. Popularity alone is therefore insufficient as a criterion for scientific engagement. Instead, future research programs should prioritize lay theories based on a combination of factors, including cultural reach, potential societal impact, relevance to core relational processes, and the downstream consequences—beneficial or harmful—of widespread adoption. Such prioritization is especially important because rigid or prescriptive adoption of lay theories can unintentionally constrain relational growth, shaping expectations about effort, obligation, and persistence in ways that discourage adaptation and change. For example, endorsing beliefs such as “love at first sight”—which frame ease and immediacy as signals of relational validity—is associated with greater likelihood of disengaging from relationships when expectations are unmet, suggesting that some lay theories may encourage premature withdrawal rather than sustained investment (Knee & Petty, 2013).

Social media platforms now function as sites of collective relationship theorizing, generating frameworks such as “the ick” (i.e., a sudden loss of romantic attraction triggered by a minor behavior), alongside micro-labels like “soft launching” (i.e., gradually revealing a new romantic partner online), “love bombing” (i.e., the rapid intensification of affection or attention to signal commitment), and “breadcrumbing” (i.e., offering intermittent signals of interest without sustained investment). These terms are embedded within broader scripts for navigating digital intimacy. These scripts include norms about response timing, visibility, and transitions between platforms, as well as practices such as navigating algorithmic dating markets, managing relationships across multiple digital platforms, and interpreting ambiguous information. Although some of these ideas have begun to attract empirical attention (e.g., “the ick”; Collisson et al., 2025; and “breadcrumbing”; Navarro & Simil, 2025), existing research has largely been descriptive, emphasizing how these ideas are experienced and who reports them. Future work could extend the generative approach outlined here by examining what the rapid diffusion of frameworks such as these reveals about unmet relational needs, sources of uncertainty, and coordination problems in an increasingly mediated relational environment.

Precisely because contemporary lay theories are culturally powerful and spread quickly, they place new demands on relationship science. Some popular labels offer genuine diagnostic insight by naming coercive or harmful dynamics (e.g., “love bombing”), whereas others risk becoming prescriptive shortcuts that encourage rapid judgment of new potential partners or premature withdrawal from relationships. Many of the behaviors these terms describe—such as intermittent attention, gradual disclosure, or sudden loss of attraction—long predate social media. What is novel is the speed and perceived authority with which digital platforms name and stabilize interpretations of these behaviors into shared scripts. For relationship science, the task is therefore not simply to validate or debunk these emerging popular theories, but to determine when they clarify relational experience, when they oversimplify it, and how they shape relationship motivation, regulation, and decision-making over time. Attending systematically to the origins, appeal, and consequences of lay theories—both offline and online—offers a principled way to decide which frameworks warrant empirical testing, translational refinement, or selective correction.

## Statements of Generality, Citations, and Positionality

This review advances a framework for using popular lay theories as translational catalysts, clarifying how they organize relational meaning and how relationship science can productively engage them to build a more rigorous and resonant science of relationships. My central claims are intended to be broadly applicable to relationship contexts in which people rely on intuitive narratives, metaphors, and informal theories to make sense of intimacy, care, and connection. At the same time, the conclusions are necessarily shaped by the scope of the literatures reviewed and by the specific case study—the love languages—that anchors the analysis. Although I emphasize throughout the review that lay theories are culturally embedded and value-laden, much of the empirical work cited here—particularly research testing the love languages, destiny beliefs, and interpersonal chemistry—draws from Western, educated, industrialized, rich, and democratic (WEIRD) samples (Henrich et al., 2010). As a result, some of the processes highlighted in this review—such as responsiveness, autonomy support, and preferences for explicit communication—may manifest differently in cultural contexts that prioritize interdependence, implicit coordination, or relational obligation. While I endeavored to address cultural variation conceptually throughout this review, empirical evidence from non-WEIRD populations remains limited and represents a critical direction for future research.

The citations in this review reflect my effort to integrate research across multiple subfields of psychology—including social, personality, clinical, and cultural psychology—and across diverse methodological traditions, including laboratory experiments, longitudinal designs, daily diaries, and experience sampling studies. Nevertheless, this review is constrained by my reliance on peer-reviewed, English-language sources and by a focus on highly visible lay theories circulating primarily in North American and Western digital contexts. Other influential relational frameworks—particularly those embedded in non-Western cultural traditions, religious communities, or non-English media ecosystems—may raise distinct questions or reveal additional translational opportunities not fully captured here. For example, in East Asian contexts such as Japan and South Korea, beliefs about blood type have historically functioned as lay theories linking biological categories to stable personality characteristics. These beliefs provide heuristics into a potential partner’s personality and shape relational evaluation by framing attraction in terms of perceived compatibility. Like the love languages, blood-type frameworks rely on categorical distinctions, but they differ in how relational change and responsibility are conceptualized. Whereas the love languages emphasize behavioral adaptation to relatively stable preferences, blood-type models tend to essentialize personal characteristics, encouraging assumptions about relational behavior that are treated as inherent rather than modifiable. This contrast illustrates how different lay theories distribute responsibility for attraction and intimacy—whether improvement is expected to arise through effortful adjustment or is accommodated as a feature of stable personal traits.

This review is shaped by my positionality as an academic psychologist trained in relationship science and situated in a research-intensive university in a Western context. My interest in lay theories is motivated by the persistent gap I have observed between how relationships are studied in academic research and how people make sense of their relational experiences in everyday life. This perspective informs my emphasis on empirical testing, construct clarity, and bidirectional translation between scientific and public discourse. Scholars from other disciplinary traditions (e.g., anthropology, communication, theology), cultural contexts, or lived experiences may approach lay theories differently, foregrounding meanings, values, or consequences on which I have placed less emphasis in this review. Engaging these perspectives alongside the present framework will be essential for extending its relevance, rigor, and cultural reach.

## Conclusion

Popular lay theories of relationships are often treated as oversimplifications to be corrected or myths to be debunked. This review advances a different perspective: lay theories persist not because they are scientifically precise, but because they resonate with people’s lived relationship experiences. Whether articulated in self-help books, therapy offices, or digital cultures, lay theories actively organize how people interpret intimacy, regulate effort and obligation, and decide what “counts” as love. Because the interpretations these frameworks promote shape whether people persist, withdraw, or attempt repair in their relationships, they also help produce the relational patterns that scientific models seek to explain. For this reason, translation becomes a substantive scientific concern rather than a secondary matter of dissemination. Just as relationships thrive when partners listen and adapt to one another, science advances when it takes seriously how people make of their own relationships and allows those meanings to inform its models. A rigorous and resonant science of relationships must therefore attend not only to what is true but to what travels. The aim is not only to better understand how love works, but to build a psychological science that works with—and for—the people it seeks to serve.
